# Natural selection and evolution: evolving concepts

**DOI:** 10.20935/AcadBiol6245

**Published:** 2024-05-27

**Authors:** Andre J. van Wijnen, Eric A. Lewallen

**Affiliations:** 1 Department of Biochemistry, University of Vermont, Burlington, VT, 05405, USA.; 2 Department of Biological Sciences, Hampton University, Hampton, VA, 23668, USA.

**Keywords:** biological law, biological theory, phenotype, genotype, fitness

## Abstract

Many recent studies in evolutionary biology have expanded and refined definitions of biological evolution and natural selection. Current evolutionary models incorporate different adaptive and non-adaptive processes based on molecular genetic changes and how DNA is modified over time in unicellular species, or in germline versus somatic cells in metazoan species. Cogent arguments can be raised for the view that natural selection should be considered a biological law, consistent with quantitative mathematical equations that describe the fitness of individuals, as well as variations within and among populations. Evolution is an overarching framework that incorporates the laws of natural selection and clarifies why phenotypic variation can increase in prevalence and result in species adaptations. The conceptual framework for biological evolution incorporates many cohesive principles that collectively have a predictive value. This framework will continue to evolve with improvements in high-resolution technologies that enable us to examine both adaptive and non-adaptive changes that drive biological phenotypes.

## 1. Introduction

Biological evolution is a cognitive abstraction that clarifies our understanding of fundamental processes at many, if not all, biological levels from molecules to species. One well-recognized mechanism by which evolution can proceed to produce biological innovations and enhance species survival is through natural selection. A useful definition for natural selection is differential survival and reproduction of individuals due to differences in phenotype, resulting in the evolution of heritable traits within a population over generations [1]. Our knowledge of biological evolution continues to advance into the third millennium. We have a deepening understanding of how DNA evolves through a range of modifications, including mutations due to replication errors, genotoxic chemicals, radiation, viral integration, and mobile DNA. A better understanding of the interplay between the environment and DNA via metabolic pathways and epigenetic mechanisms that control the accessibility of genetic information has further contributed to fundamental conceptualizations of how evolution works.

As novel insights are obtained with innovative experimentation and newer technologies, many classical debates have become outdated. Classical philosophical discussions between the importance of ‘nurture and nature’ in evolution (Lamarckism versus Darwinism) promulgate a false distinction because genetics, random somatic events, and environmental factors collectively contribute to the relative fitness of individuals within a species and population. A recent paper by Crouch and Bodmer poses that *evolution by natural selection is a scientific law and not a scientific theory*. Scientific terms like ‘theory’ and ‘law’ are absolute words with deep philosophical roots [2]. The exact meaning of these two words may depend on linguistic interpretations from British, American, or international perspectives. Whether evolution as a conceptual framework is a ‘theory’ or a ‘law’ is only meaningful to those who appreciate the distinction in their definition, which may elude many. Crouch and Bodmer, who are from Oxford University, quote from the Oxford English Dictionary that ‘Law, n.’ is “*a theoretical principle deduced from particular facts*” and that it has predictive powers. Consistent with that terminology, the term ‘law’ in a scientific context refers to ‘small’ ideas embodied by a single equation, and theories refer to larger bodies of ideas. Yet, Crouch and Bodmer pose that evolution (by natural selection) is a law because it is an inevitable consequence of the nature of all living organisms, while mathematical equations in physics are theories. In other academic circles, this type of phrasing simply creates confusion in broadly accepted terminology and is a reflection of how language and human understanding of complex concepts evolves differently in distinct groups.

A commonly accepted understanding of the phrase ‘scientific law’ is that it typically refers to precise quantitative rules. For example, Newton’s and Einstein’s Laws clarify with quantitative precision how inanimate matter and energy behave. Scientific theories provide broad concepts with overarching explanations of how nature works (e.g., the Big Bang theory incorporates physical laws). From this perspective, equating the mechanism of natural selection with evolution is a *pars pro toto* and calling evolution a law represents a *toto pro pars* in linguistic terms.

### Pragmatic view of evolutionary processes.

Biological evolution is based on a comprehensive, cohesive, and consistent set of qualitative and quantitative facts that conform to specific mathematical equations (‘laws’: e.g., Mendelian laws, natural selection laws). Natural selection specifically is a biological law that can be captured in defined equations as discussed by Crouch and Bodmer [2]. The broad impact of evolution goes beyond these equations and is qualitatively evident in the properties of DNA as a palimpsest in any phylogenetic comparison with both meaningful and meaningless changes in DNA sequences from a fitness perspective. Collectively, biological evolution is an overarching concept that incorporates a multitude of facts and laws validated across multiple scientific subdisciplines. This cross-disciplinary validation reinforces the tremendous utility of evolution as an essential idea that explains much of the animate world. Evolution is a powerful utilitarian principle that makes sense of our biological world, regardless of whether it fits the absolute definition of a ‘theory’ or a ‘law’. However, because evolution is a broader concept than the more focused biological scientific laws that support it, for the sake of the argument, one could favor the phrasing that evolution is in fact a theory.

Crouch and Bodmer describe ‘theory’ as a set of logical relations in natural systems that lead to predictable and experimentally verifiable outcomes [2]. However, their concern is that a scientific theory is never guaranteed to be true, because there could be competing theories. They note historical disagreements over the fundamental validity and explanatory power of the laws that describe evolution, based on the circular argument (i.e., ‘tautology’ in their phrasing) that organisms that reproduce the most are those that are reproductively most successful [2]. Here, the authors justifiably conclude that natural selection is a law and not a scientific theory. One main point advanced by Crouch and Bodmer is that the term ‘law’ reinforces the idea that natural selection is an inevitable consequence of life, while the potentially pejorative label ‘theory’ could easily create uncertainty about the inevitability of natural evolution. Others may firmly believe in the power of natural selection, but may consider the process not necessarily ‘inevitable’. It is hard to prove such absolute terms because it requires definitive proof that the statement is true in all conceivable circumstances. This can be done with mathematical equations but not with broad biological concepts. Therefore, it is equally acceptable for individuals to remain agnostic of the inevitability of natural selection, while still being certain that it definitely influences the history, present, and future of life.

In recent years, a broader discussion on the topic of evolution and natural selection has emerged as a large body of work that provides contemporary perspectives [3–42]. The latter studies collectively discuss the range of roles of function and selection in evolving systems, such as non-genetic inheritance, heredity of acquired characteristics, the relative contributions of genetic versus non-genetic transmission of heritable information, and genetic drift, gene flow, and mutational variations as pre-conditions of evolution [3–39]. Other authors emphasize the urgent need to develop integrated models for the multiple modes of [40], as well as to differentiate between, stabilizing and directional selection [41, 42]. Evolution may emerge as a consequence of at least two different types of mechanisms: deterministic, or non-random (i.e., selection) and stochastic mechanisms (i.e., mutation, re-combination, and genetic drift). Furthermore, natural selection is confounded by sexual selection that can lead to non-adaptive traits, artificial selection, group and/or kin selection, and multi-level selection.

Crouch and Bodmer note that most of evolutionary history occurred in prokaryotic microbes, which do not recombine and have very large population sizes, indicating that mutation has been the principal factor among these various stochastic mechanisms for much of evolutionary history. Furthermore, they note that neutral alleles (natural sequence variation in non-coding regions) typically have little or no phenotypic effects, and are irrelevant to evolution of organisms by natural selection. Rather, they pose that non-coding sequences (e.g., repetitive sequences such as Richard Dawkin’s “selfish genes”) have their own molecular evolution by natural selection. It should be noted that this class of repetitive sequences are typically transposons that resemble endogenous integrated viruses and represent only a fraction of non-coding sequences.

In their opinion, Crouch and Bodmer emphasize that the term ‘law’ for evolution by natural selection is most appropriate, but not because natural selection can be described mathematically. They believe that *natural selection is different from scientific theories because the conditions are very highly evidenced (effectively proven) by the nature of all living organisms, whereas in a theory it is only ever the outcomes that are evidenced*. One compelling alternative view is that natural selection is a law, precisely because it can be captured in exact equations that can be mathematically validated for all input variables from zero to infinity.

## 2. Conclusions

Natural selection as a law is not the same as evolution as a concept. Natural selection is a quantifiable law and captured by mathematical equations, while evolution is a solid theory that applies this law. From a molecular perspective, phylogenomic analysis of non-coding DNA shows that DNA evolves independently of natural selection. The vast majority of DNA is non-coding. Hence, at the molecular level, there is a neutral evolution of DNA sequences, and therefore, biological evolution of DNA is not equivalent to natural selection. Crouch and Bodmer conclude rather firmly that “adaptive evolution by natural selection is a scientific law” [2]. An alternative conclusion would be that biological evolution is a fundamentally sound concept that encompasses both adaptive and non-adaptive processes, while natural selection is a quantitative mathematical law that clarifies adaptive evolution. The thoughts and conclusions expressed by many investigators [1–42] indicate that evolutionary biology remains a dynamic field that continues to refine mechanistic models by which adaptive evolution drives species innovation in parallel to non-adaptive evolutionary processes that generate variation that is not of immediate utility.

## Data Availability

Data supporting these findings are available within the article, at https://doi.org/10.20935/AcadBiol6245, or upon request.
